# Positional Change of the Eyeball During Eye Movements: Evidence of Translatory Movement

**DOI:** 10.3389/fneur.2020.556441

**Published:** 2020-09-17

**Authors:** Yeji Moon, Won June Lee, Seung Hak Shin, Ji Hong Kim, Ji Young Lee, Sei Yeul Oh, Han Woong Lim

**Affiliations:** ^1^Department of Ophthalmology, Hanyang University Hospital, Hanyang University College of Medicine, Seoul, South Korea; ^2^Department of Ophthalmology, Armed Forces Capital Hospital, Seongnam, South Korea; ^3^Department of Radiology, Hanyang University Hospital, Hanyang University College of Medicine, Seoul, South Korea; ^4^Department of Ophthalmology, Samsung Medical Center, Sungkyunkwan University School of Medicine, Seoul, South Korea

**Keywords:** translation, eyeball movement, three-dimensional magnetic resonance imaging, strabismus, translatory movement

## Abstract

**Purpose:** To investigate the positional change of the eyeball induced by horizontal and vertical gazing to deduce translatory movement, using three-dimensional (3D) magnetic resonance imaging (MRI).

**Methods:** In this prospective observational study participants underwent orbital MRI during central, right, left, up, and down gazing. MRI scans were processed using self-developed software; this software enabled 3D MR image reconstruction and the superimposition of reconstructed image sets between different gazes. After acquiring the coordinates of the eyeball centroid in each gaze, the changes in centroid coordinates from central gaze to the other gazes were estimated, and correlations with associated factors were evaluated.

**Results:** The mean distance of centroid movement was 0.69 ± 0.27 mm in abduction, 0.68 ± 0.27 mm in adduction, 0.43 ± 0.23 mm in elevation, and 0.44 ± 0.19 mm in depression. The mean angle of centroid movement in horizontal gaze, measured in terms of the movement of the left eye centroid in the axial plane, was 228.7° in abduction and −4.2° in adduction. In vertical gaze, the mean angle of centroid movement was −96.8° in elevation and 101.8° in depression. Axial length and ocular volume were negatively correlated with the distance of centroid movement in horizontal gaze.

**Conclusions:** The position of the eyeball moved in the same direction as the gaze during horizontal gaze, but in the opposite direction during vertical gaze. For accurate eye movement analyses, such as the measurement of the deviation angle in strabismus, translation should be considered in addition to rotation.

## Introduction

The complex three-dimensional (3D) movements of the eyeball are mediated through the action of the extraocular muscles (EOMs) in the orbit (1, 2). There are six EOMs responsible for eye movements about three axes of rotation: abduction and adduction; elevation and depression; and intorsion and extorsion. While the major eye movement is rotation, other movement patterns are also mechanically possible because the eyeball is partly free to move in the orbit. The eyeball is surrounded by soft tissues, including orbital fat, and is not fixed to a shaft-like structure that forms a fixed axis of rotation (3). Therefore, positional change of the eyeball itself, termed translatory movement, can occur within the orbit.

Eye movements have been studied for many years, and references to translatory movement have been made since the nineteenth century (4). However, owing to the assumption that ocular translation is negligible and rarely occurs, the basic laws of eye movement only take into account rotation on a fixed axis through the center of the eyeball (5–7). Therefore, despite a general acknowledgment of its occurrence, there are few studies of ocular translation (8).

Technical advancements in magnetic resonance imaging (MRI) now enable the in-depth and non-invasive study of eye movement in living subjects (9, 10). MRI provides high soft tissue contrast along with high spatial resolution in multiple planes (11), enabling the anatomical structure of the orbit to be visualized in detail. Therefore, MRI has contributed to the investigation of functional eyeball position and the determination of the effect of EOMs during gaze shifts (12). Moreover, the digital reconstruction of MRI images enables the analysis of complex 3D eye movements during visual gaze (13).

We recently described translatory eye movements using the 3D reconstruction of MRI images (14). In this study, translatory movement of the eyeballs toward the direction of gaze was observed directly in superimposed 3D images. Based on this study, we used high-resolution MRI to investigate the patterns of eye movement in normal subjects during gaze shifting. The aims of the present study are to investigate the positional changes of the eyeball during gaze shifting in order to deduce its translatory movement, and to identify correlations between translatory movement and associated factors including axial length, ocular volume, and orbital volume.

## Methods

This study was approved by the Institutional Review Board of Hanyang University Hospital, and followed the tenets of the Declaration of Helsinki for biomedical research. The study was fully explained to all participants, and informed consent was obtained prior to their inclusion in the study. To ensure that the subjects did not suffer from any ophthalmic disease, a comprehensive ophthalmologic examination was performed including best-corrected visual acuity, tonometry, refraction, slit-lamp examination, dilated funduscopic examination, and ocular motility testing. Axial length was measured using partial coherence interferometry (IOLMaster 500; Carl Zeiss Meditec, Zeiss Humphrey System, Dublin, CA). Provided that the ophthalmologic examination revealed no ocular abnormality, each subject underwent high-resolution, T2-weighted orbital MRI using a 3.0 T whole-body scanner (Achieva 3.0T; Philips Medical Systems, Best, The Netherlands) with a 32 channel head coil. Ocular and orbital volumes were measured using Voxar software (Toshiba Medical Visualization Systems). Exclusion criteria were as follows: (1) any retinal or optic nerve disorders identified on the comprehensive ophthalmologic examination, (2) any abnormal findings on MRI, (3) any ocular motility dysfunctions found on physical examination, (4) a history of ophthalmologic or neurologic diseases, and (5) previous ocular or periocular surgery.

### Image Acquisition Procedures

We obtained T2-weighted MRI images (repetition time = 2,500 ms; echo time = 248 ms; flip angle = 90°; section thickness = 0.6 mm; field of view = 180 × 180 mm; matrix = 256 × 256; voxel size = 0.7 × 0.7 × 0.6). To protect their hearing, subjects wore earplugs during the session. Subjects were placed in a supine position and their heads were carefully stabilized by securely fastening the head coil mask to the face with bands, and fixing the mask to the scanner gantry with foam cushions and tapes. There was an opening in the head coil mask above the eyes such that subjects could fixate on targets through this open space. Fixation targets were secured on the inside of the scanner bore; the bore diameter was 60 cm. As the scanner bore was always in the same position during imaging, the fixation target points were presented in an identical fashion to all subjects.

**Figure 1:**
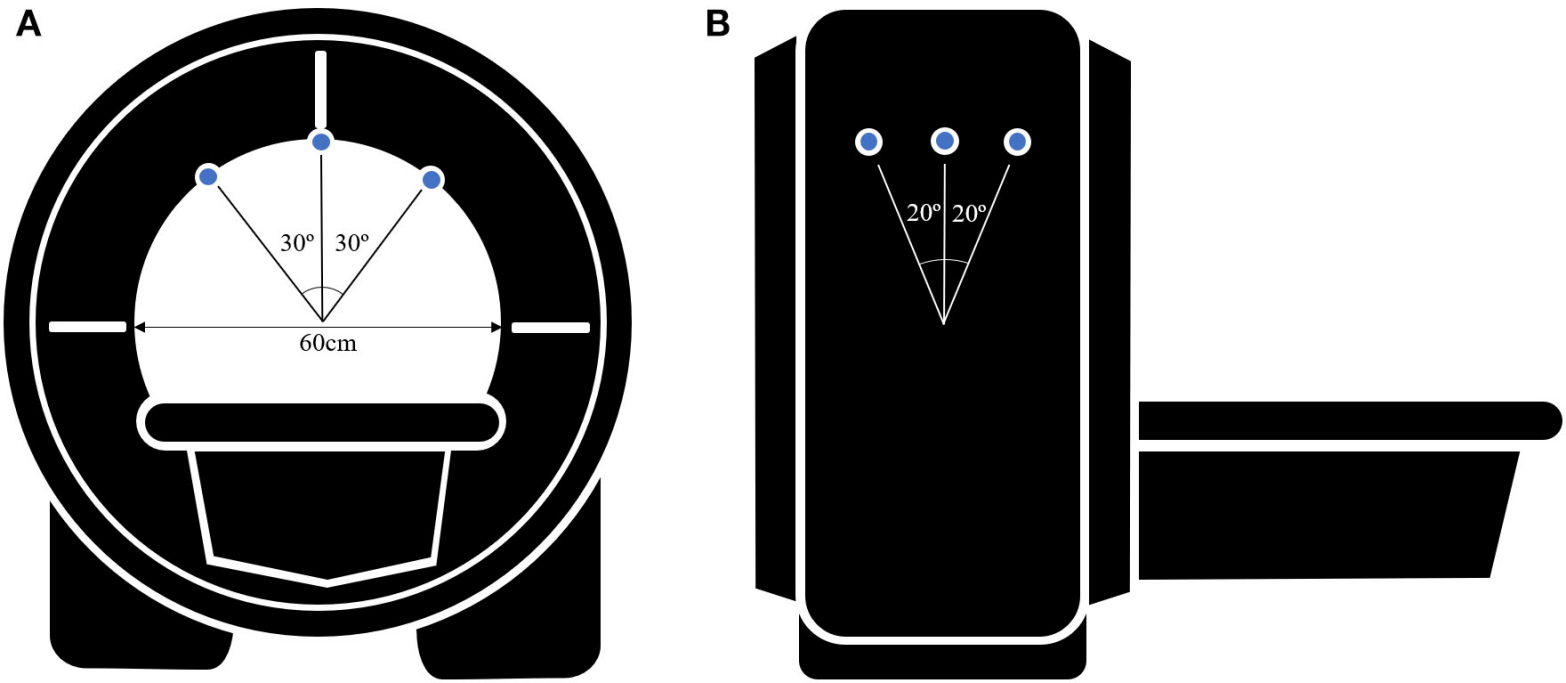
Schematic figure of the MR images acquisition setup **(A)** in a axial view and **(B)** in a sagittal view. Small blue circles indicate the fixation targets. Fixation targets are on the inside of the scanner bore and the bore diameter is 60 cm. Fixation targets are placed at a 30° angle of the gaze when assessing horizontal movements and a 20° angle of the gaze when assessing vertical movements.

Initially, a low-resolution triplanar scan was performed to inform the localization of subsequent imaging. Sets of 67 contiguous, 0.6 mm-thick quasi-transverse images were obtained, occupying a 40.2 mm field of view. Scanning was repeated when patients were looking at the appropriate fixation target for each gaze: central gaze, right gaze, left gaze, upgaze, and downgaze. Fixation targets were placed at a 30° angle of the gaze when assessing horizontal movements and a 20° angle of the gaze when assessing vertical movements, based on former studies (15, 16). (Figure 1) For all orbital MRI scans in this study, the same imaging design, quasi-transverse imaging planes, fixation targets, and gaze directions were used.

### 3D MRI Processing Procedures

It should be noted that the strategy we used to fix the patients' heads was insufficient to minimize error in evaluating eyeball positional changes, because the head was able to move slightly when the eyeballs moved. Hence, before analyzing the images, it was necessary to three-dimensionally match the position of the static tissues other than the eyeball in the MRI images for all gazes.

**Figure 2:**
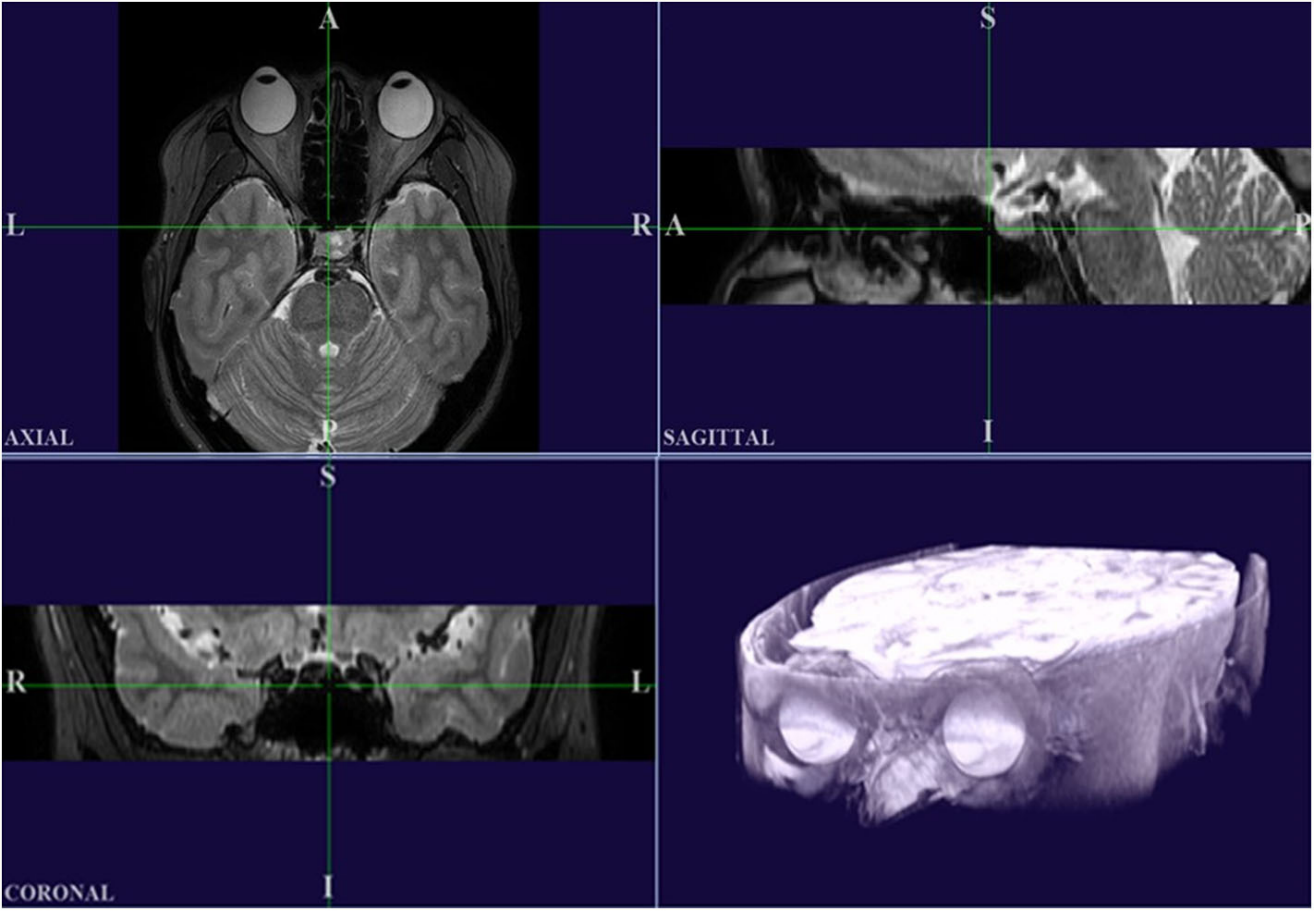
High-resolution MRI in multiple planes, and a 3D reconstructed image produced using our custom Visual C++ program. It should be noted that the axial plane in this program is inverted left and right compared to the original MRI.

For this process, digital MR images were converted into a DICOM file format and images were processed using custom analysis programs written in Visual C++ using Visual Studio Community software (Version 2015; Microsoft, Redmond, WA, USA). Then MR images of the primary and secondary positions were three-dimensionally reconstructed using our program. Axial, sagittal, and coronal images were used for 3D reconstruction (Figure 2). This software was designed to ensure accurate superimposition. MR images of the secondary positions were adjusted based on the position of the static tissues in the MR image of the primary position. Two authors (JK and HL) independently checked the axial, sagittal, and coronal images to see if the alignment was correct and check whether any structures other than the eyeball showed movement between the images. When both authors agreed that the MR images were well-aligned, the image analysis for the centroid coordinate extraction could proceed. To clarify this procedure, a video component is attached to the Supplementary Material. Then, to enable measurement of the degree of eyeball movement, we stored the images of the primary and secondary positions separately.

### Image Analysis

**Figure 3:**
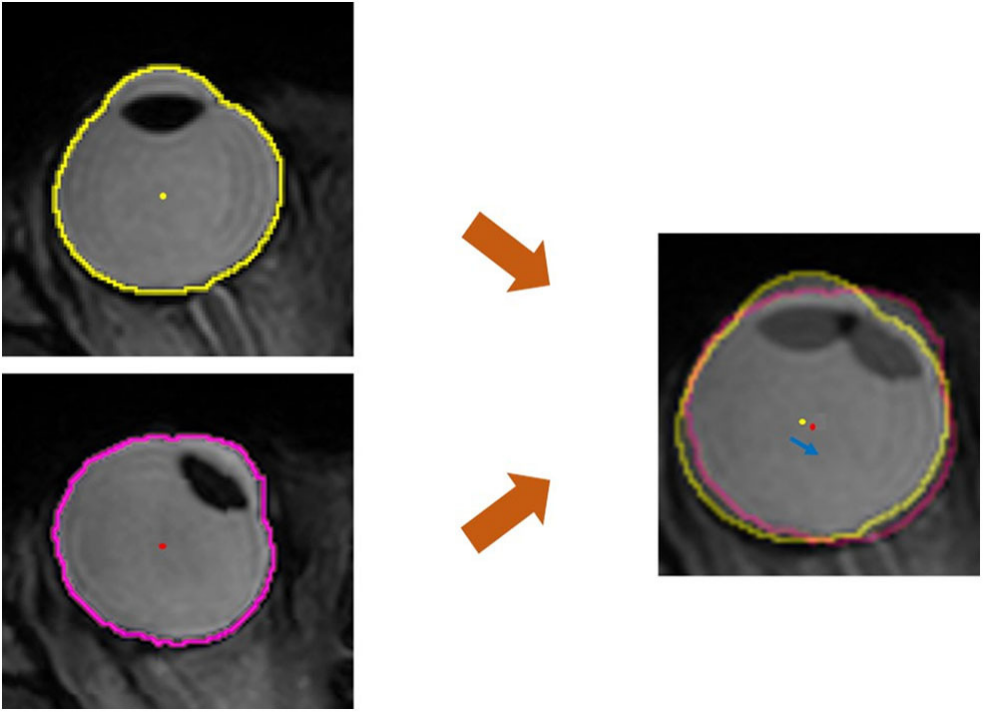
Quantitative measurement of the movement of the eyeball centroid. For the primary position (central gaze), the extracted outline of the eyeball image is marked with a yellow line and the centroid of the eyeball is marked with a yellow dot. In horizontal gaze, the eyeball outline is marked with a pink line and a red dot indicates the centroid of the eyeball. The centroid, which is the geometrical center of the extracted eyeball, was automatically obtained in the form of x, y, and z coordinates. In the superimposed image, which was adjusted using static tissues, the distance of centroid movement was obtained using the distance formula, and the direction of centroid movement was calculated with the arctangent formula. The blue arrow indicates the movement of the centroid.

**Figure 4:**
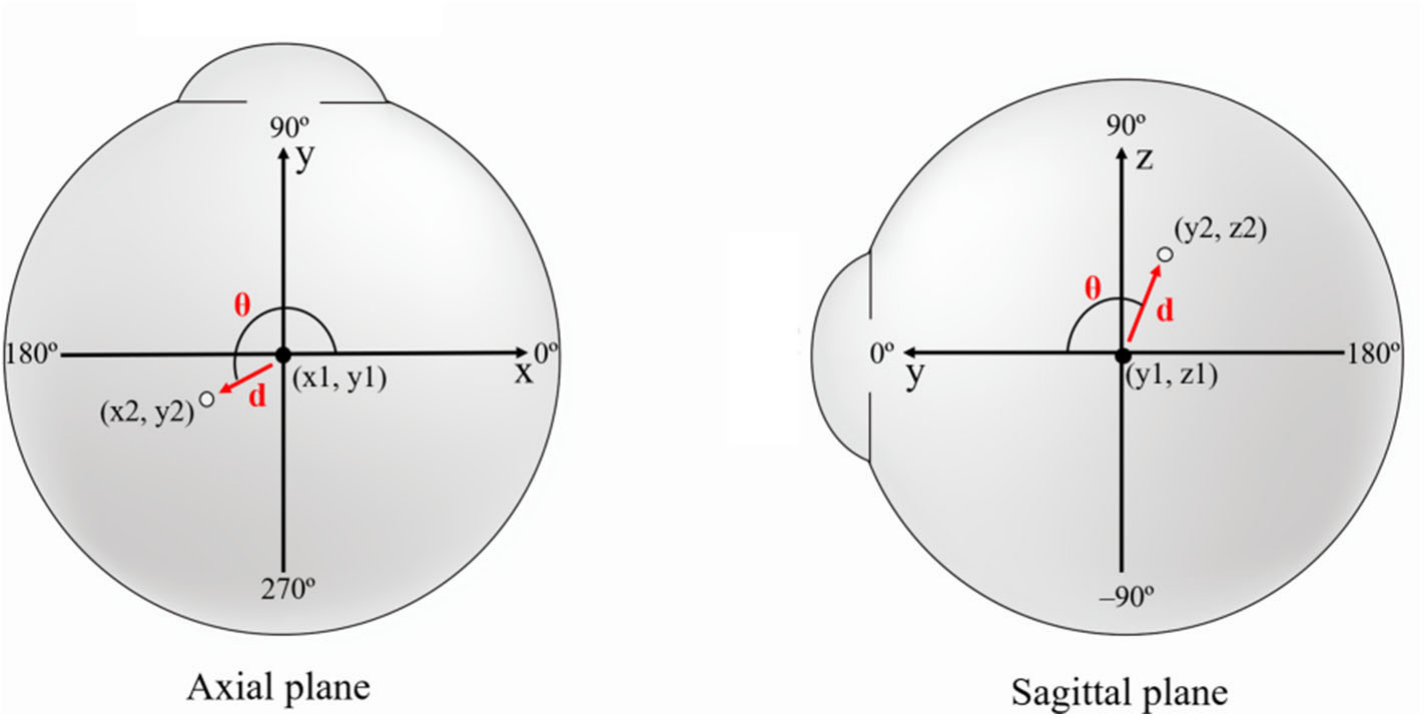
Schematic figure showing the values used in image analysis. To evaluate the positional change of the eyeball, we measured the distance (*d*) and direction (θ) of eyeball centroid movement between the primary position (black dot) and each secondary position (white dot). In horizontal gaze, axial plane (X-Y plane) images were used and the appropriate x and y coordinates were calculated to determine the distance and direction of eyeball translation. In vertical gaze, sagittal plane (Y-Z plane) images were used, and y and z coordinates were calculated. The red arrow indicates centroid movement.

To evaluate the positional change of the eyeball, we measured the distance and direction of movement of the eyeball centroid between the primary position and each secondary position. The centroid of any plane figure is the arithmetic mean position of all the points in the shape. Therefore, the centroid represents the eyeball position as long as the eyeball shape does not change. We determined the position of the centroid in order to calculate the amount of eyeball movement (Figure 3). First, the contour of the eyeball was extracted using the ImageJ (National Institutes of Health, Bethesda, MD, USA) wand (tracing) tool. After the wand tool marked the eyeball contour, the ImageJ program measured the centroid, yielding x, y, and z coordinates. All the image analysis procedures were automatically conducted. In the 3D images, the three axes were the lateral-medial axis (x-axis), antero-posterior axis (y-axis), and vertical axis (z-axis). To assess horizontal movement, axial plane (X-Y plane) images were used and the appropriate x and y coordinates were calculated to determine the distance and direction of eyeball translation. To assess vertical movement, sagittal plane (Y-Z plane) images were used, and y and z coordinates were calculated (Figure 4).

The distance of centroid movement was calculated using a distance formula. As the ImageJ program used the pixel as a standard unit, we converted pixels to millimeters. The movement direction of the centroid was calculated using an arc-tangent formula, yielding angles in degree units. In the analysis of horizontal gaze, the following formulae were used:

Distance formula

$$\begin{array}{l} \text{d }\left(\text{mm}\right)=\sqrt{{\left(x2-x1\right)}^{2}+{\left(y2-y1\right)}^{2}} \end{array}$$

Arc-tangent formula

$$\begin{array}{l} \text{θ}{(}^{\circ })=\frac{{\text{tan}}^{\text{-1}}\left\{\left(y2-y1\right)/\left(x2-x1\right)\right\}}{\pi }\times 180 \end{array}$$

d: distance between centroid positions in central and horizontal gaze, θ: angle of the direction of movement of the centroid from central to horizontal gaze, *x*1 and *y*1: x and y coordinates of the centroid position in central gaze, *x*2 and *y*2: x and y coordinates of the centroid position in horizontal gaze.

In horizontal gaze, an additional transformation was required for the arc-tangent formula to analyze the direction of movement of the left and right eyes together. Since adduction or abduction of the right and left eyes is symmetric about the y-axis, the x-coordinate shift of the centroid measured in the right eye (*x*2−*x*1) was multiplied by −1 and used in the arc-tangent formula to match the left eye measurement.

Meanwhile, the following formulae were used for the analysis of vertical gaze:

Distance formula

$$\begin{array}{l} \text{d }\left(\text{mm}\right)=\sqrt{{\left(y2-y1\right)}^{2}+{\left(z2-z1\right)}^{2}} \end{array}$$

Arc-tangent formula

$$\begin{array}{l} \text{θ }{(}^{\circ })=\frac{{\text{tan}}^{\text{-1}}\left\{\left(z2-z1\right)/\left(y2-y1\right)\right\}}{\pi }\times 180 \end{array}$$

d: distance between centroid positions in central and vertical gaze, θ: angle of the direction of movement of the centroid from central to vertical gaze, y1 and *z*1: y and z coordinates of the centroid position in central gaze, *y*2 and *z*2: y and z coordinates of the centroid position in vertical gaze.

### Statistics

We used SPSS (version 17.0; SPSS Inc., Chicago, IL, USA) for all our analyses. One eye of each subject was randomly selected for statistical analysis by alternately selecting right and left eyes from the randomly ordered sample. All axial plane images were aligned for analysis based on the left eye's orientation. In the axial plane relative to the left eye, medial was defined as 0°, anterior as 90°, lateral as 180°, and posterior as 270° counterclockwise. In the sagittal plane for the vertical gaze, anterior was defined as 0°, upwards as 90°, and downwards as −90°. The independent samples Student's *t*-test was used for comparisons between gaze directions. Pearson's correlation coefficient was calculated to estimate the correlations between the distance of centroid movement and associated factors including axial length, eyeball volume, and orbit volume. A *p* ≤ 0.05 was considered statistically significant. All data were presented as mean ± standard deviation.

## Results

Fifty-six subjects (33 males and 23 females) were included in this study. The mean age of all subjects was 31.1 ± 9.6 years (range: 20–50 years). Mean axial length was 25.27 ± 1.71 mm (range: 21.22–29.52 mm) and mean ocular volume was 7.51 ± 1.17 mm^3^ (range: 5.11–11.02 mm^3^). Mean orbital volume was 26.0 ± 3.2 mm^3^ (range: 20.84–32.82 mm^3^). The mean angle of eyeball rotation for each gaze was 33.3 ± 6.2° in abduction, 34.5 ± 8.0° in adduction, 20.1 ± 4.8° in elevation, and 23.4 ± 3.1° in depression.

### Distance and Direction of Centroid Movement

**Figure 5:**
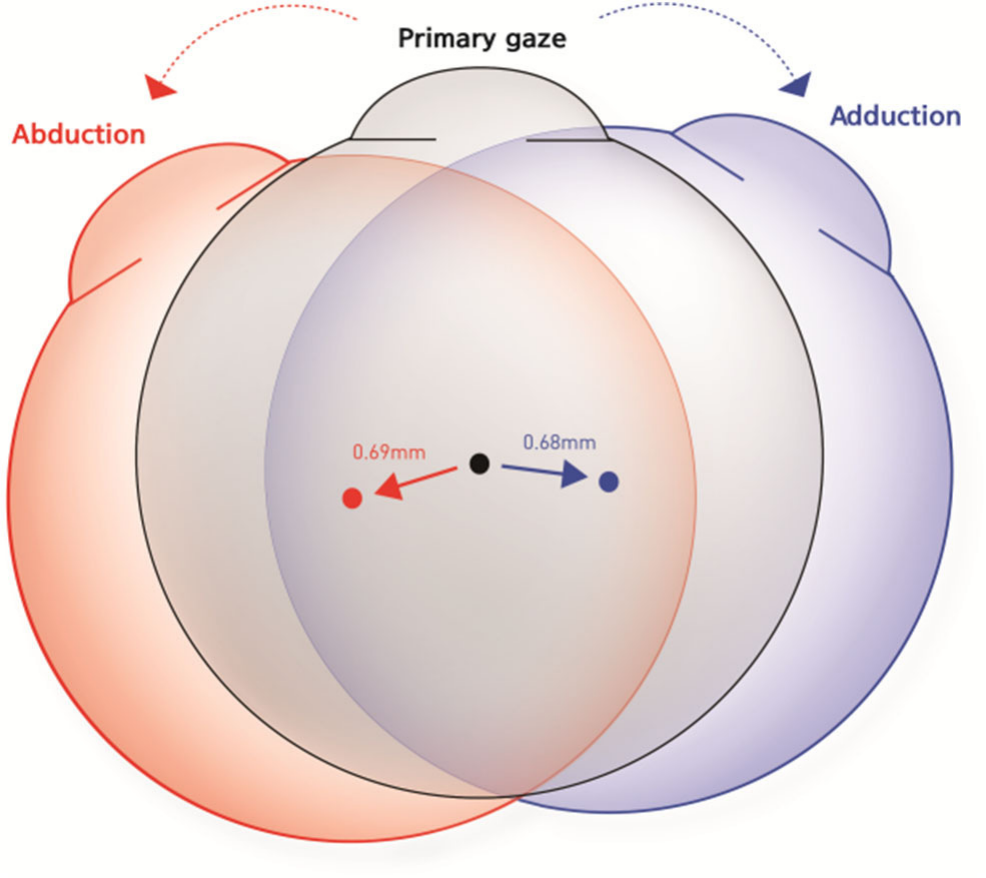
Schematic figures to represent the mean distance and direction of centroid movement in horizontal eye movements. The mean distance of centroid movement was 0.69 ± 0.27 mm in abduction and 0.68 ± 0.27 mm in adduction. The eyeball moved in the direction of the gaze during horizontal gaze.

Although there were individual differences, all subjects exhibited positional change of the eyeball in all gaze directions. The mean distance of centroid movement in horizontal gaze was 0.69 ± 0.27 mm (95% confidence interval [CI]: 0.63–0.75 mm) in abduction and 0.68 ± 0.27 mm (95% CI: 0.62–0.74 mm) in adduction (Figure 5). In vertical gaze, the mean distance of centroid movement was 0.43 ± 0.23 mm (95% CI: 0.37–0.49 mm) in elevation and 0.44 ± 0.19 mm (95% CI: 0.39–0.49 mm) in depression. There were no significant differences in the distance of centroid movement between abduction and adduction (*p* = 0.870) or between depression and elevation (*p* = 0.700).

**Figure 6:**
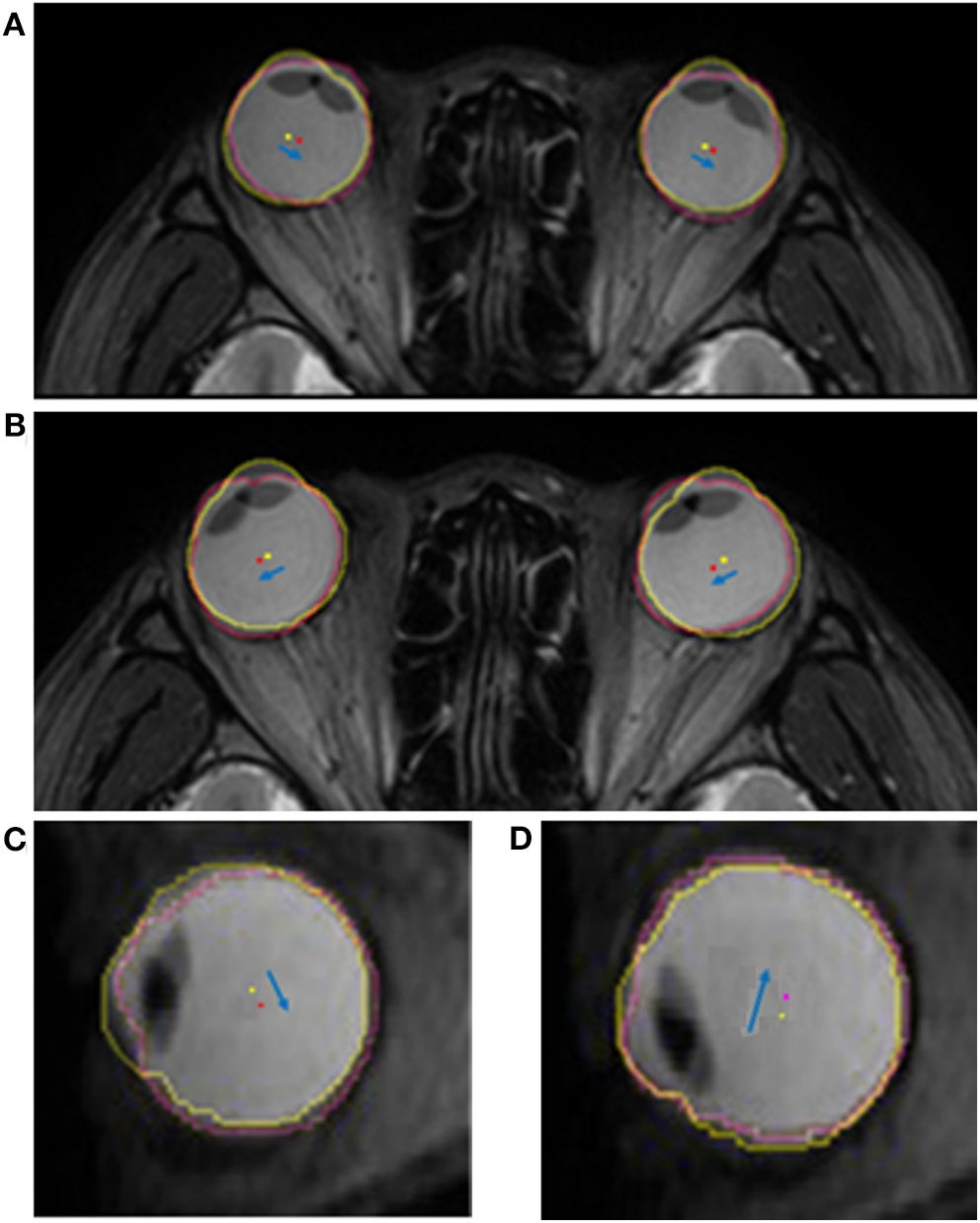
Superimposed MR images showing the translatory movements of the eyeball. The yellow and red dots indicate the centroid of the eyeball in the primary and secondary positions, respectively. The blue arrow indicates the movement of the centroid of each eyeball. Eyeballs move toward the gaze direction in the axial plane during horizontal gaze. **(A)** When the subject looks to the right, the centroids of both eyeballs translate to the right. **(B)** Similarly, when the subject looks to the left, the centroids of both eyeballs translate to the left. The translatory movement of the eyeball in the sagittal plane is in the opposite direction to the gaze during vertical gaze. **(C)** The eyeball is translated downwards when looking upwards, and **(D)** translated upwards when looking downwards.

The eyeball moved in the direction of the gaze during horizontal gaze; thus, the eyeball position moved outward in abduction and inward in adduction. The mean angles of direction, in terms of the movement of the left eye centroid in the axial plane, were 228.7 ± 4.0° in abduction and −4.2 ± 3.1° in adduction. Conversely, the centroid moved in the opposite direction to the gaze in vertical gaze; thus, eyeballs moved upwards in downgaze and downwards in upgaze. The mean direction was −96.8 ± 3.4° in upgaze and 101.8 ± 5.8° in downgaze in the sagittal plane (Table 1, Figure 6).

**Table 1:** The mean distance and direction of centroid movement in different gazing direction.

	**Distance (mm)**	**Direction (**°**)**
Abduction	0.69 ± 0.27 (0.63–0.75)	228.7 ± 36.4° (220.7–236.7)
Adduction	0.68 ± 0.27 (0.62–0.74)	−4.2 ± 26.5° (1.24–10.9)
Elevation	0.43 ± 0.23 (0.37–0.49)	−96.8 ± 26.2° (−90.1–103.5)
Depression	0.44 ± 0.19 (0.39–0.49)	101.8 ± 46.2° (90.1–113.4)

*Data are mean ± standard deviation (95% confidence interval). The direction of translation was calculated according to the left-eye orientation*.

### Correlations Between Centroid Movement and Ocular Biometry

**Figure 7:**
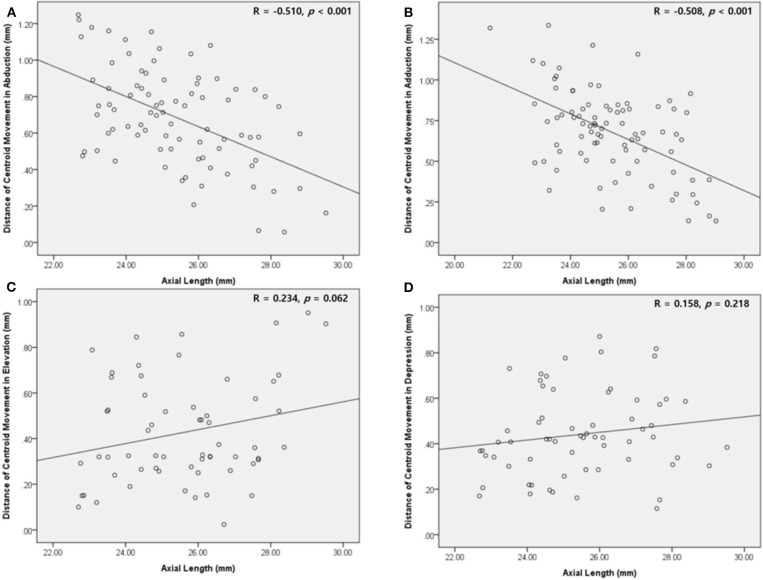
Scatter plot showing the correlation between the mean distance of the centroid movement and the axial length. **(A,B)** In abduction and adduction, the length of centroid movement and the axial length are negatively correlated. **(C,D)** In elevation and depression, the length of centroid movement showed insignificant correlation with the axial length.

Axial length was negatively correlated with the distance of centroid movement in horizontal gaze (Figure 7). Ocular volume also showed negative correlation with the distance of centroid movement. Meanwhile, the distance of vertical movement was not correlated with axial length or ocular volume. There were no significant correlations between the degree of centroid movement and orbital volume (Table 2).

**Table 2:** The correlation between the length of translatory movement and ocular biometry.

	**Axial length**		**Intraocular volume**		**Orbital volume**	
	***R***	***p*-value**	***R***	***p*-value**	***R***	***p*-value**
Abduction	−0.510	**<0.001**	−0.382	**<0.001**	0.053	0.632
Adduction	−0.508	**<0.001**	−0.428	**<0.001**	−0.057	0.604
Elevation	0.234	0.062	0.246	0.056	0.028	0.833
Depression	0.158	0.218	0.189	0.139	0.139	0.278

*Numbers in bold indicate statistically significant correlation*.

## Discussion

In this study, we employed high-resolution MRI to investigate the patterns of eye movement using the coordinates of the centroid of the eyeball. The centroid moved in the same direction as the gaze during horizontal movement, whereas it moved in the opposite direction during vertical movement. Additionally, the size of the eyeball was negatively correlated with the distance of centroid movement during horizontal gaze. Our study is novel in that we visualized the positional change of the eyeball during eye movement through 3D MRI, analyzed this movement with respect to gaze direction, and determined the correlation between the extent of movement and eyeball size.

The eye is suspended in the orbit surrounded by soft fat pads; this allows it to translate to some extent, as one can verify by pushing on the eye through the eyelid (3). However, most studies of eye movement have only examined rotational movements about a fixed center of rotation. Previous studies have reported that the eyeball can be well-approximated as a spherical joint with its center fixed in the head, such that only rotations around three orthogonal axes passing through the center of the eye need to be considered (7). Von Noorden also concluded that translatory movements may be disregarded from a practical standpoint (5). Unfortunately, despite acknowledging the occurrence of eyeball translation, they assumed that it is a negligible part of eye movement; thus, most researchers have studied only the rotational components of the entire eye movement pattern. The advantage of this assumption is that it is easier to approach eye movement and to apply basic physiological laws of motion to the complex ocular kinematics. However, our results show that the eyeball not only rotates, but also changes in position. We demonstrated a significant change in the coordinates of the centroid of the eyeball in all gaze directions. These findings suggest that the movement of the eye, like other rigid bodies, is dictated by the basic kinetics of rotational and translatory movement.

With its increasing applicability and enhanced usability, advanced eye tracking has become easier to integrate into research and clinical practice by eliminating barriers such as size and portability while maintaining high quality (17). In response to recent advancements, further understanding of eye movement is required for upgrading eye-tracking technology. Therefore, it is necessary to measure eye movements with higher accuracy and greater precision. Most eye-gaze tracking technologies rely on some properties or characteristics of the eye that can be detected and tracked by a device (4, 18) The pupil (19–22), limbus (15, 23), corneal reflex (24), and Purkinje reflex (17, 25–27) are common features used for tracking. These eye-tracking techniques also assume that eye movement only involves rotational motion, making intrinsic errors inevitable because real eye movement is composed of both rotational and translatory motion. Even though translatory movement accounts for a smaller portion than rotational movement, it should be considered in evaluating eye movement in neuroscience and clinical research.

**Figure 8:**
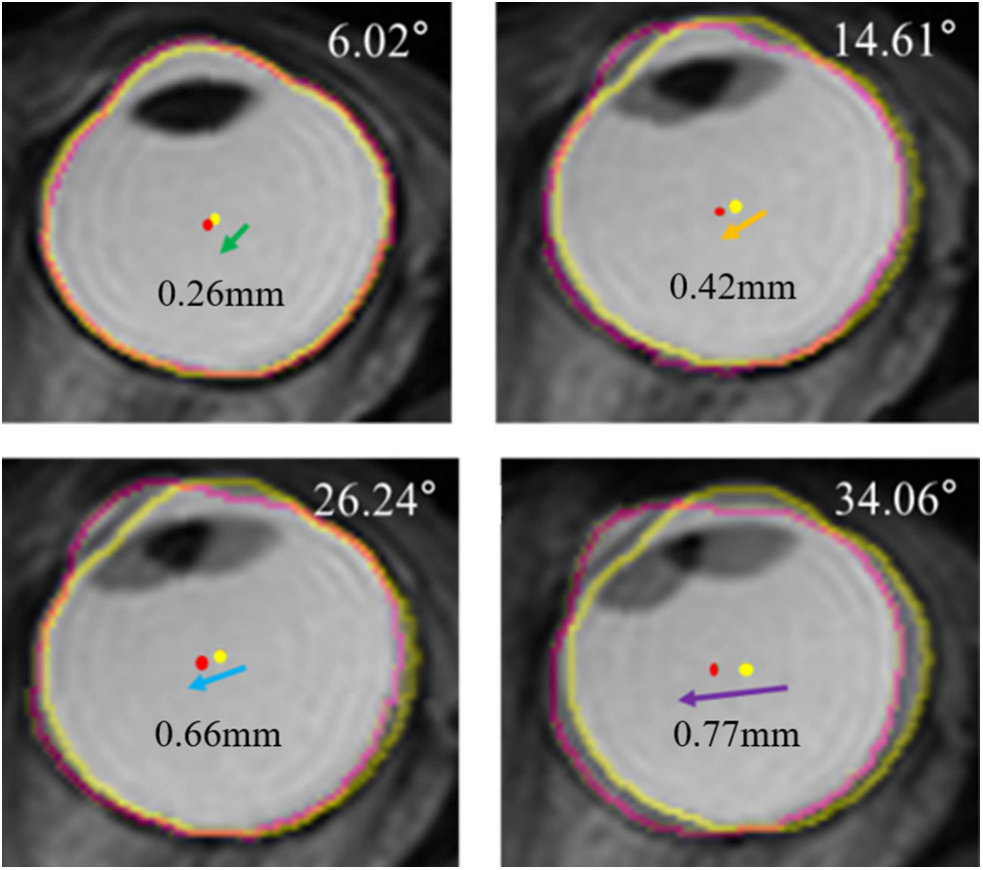
Serial superimposed MR images of right eye in a gradual increase of eyeball rotation. In a pilot study, one subject's MRI were obtained by increasing the angle of adduction gradually. After the identical processing, the extracted outline of the eyeball image is marked with a yellow line in central gaze and a pink line in left gaze. The centroid of the eyeball is marked with a yellow dot in central gaze and a pink dot in left gaze. Superimposed images show translations of eyeball, and the distance of translation increases when the rotation angle increases.

Previously translatory movements of the eyeball were thought to be observed only under unusual conditions like extreme lateral gazing, because the generation of extraocular muscle force is highest. Thus, to determine the existence of translatory movement at small angles of eye movement, we conducted a pilot study involving two normal subjects. One subject's MRI images were obtained from central gaze to right gaze by increasing the angle of eye movement by about 10° and MRI images of the other subject were obtained from central gaze to left gaze in the same manner. After the same image processing procedure that we have described above, we observed translation of the eyeball even with small eye-movement angles, such as 10° or 20° (Figure 8). These findings indicate that translation occurs not only with extreme large-angle gazes but also with small-angle gazes, which implies ocular translation should be considered for even normal eye movements.

**Figure 9:**
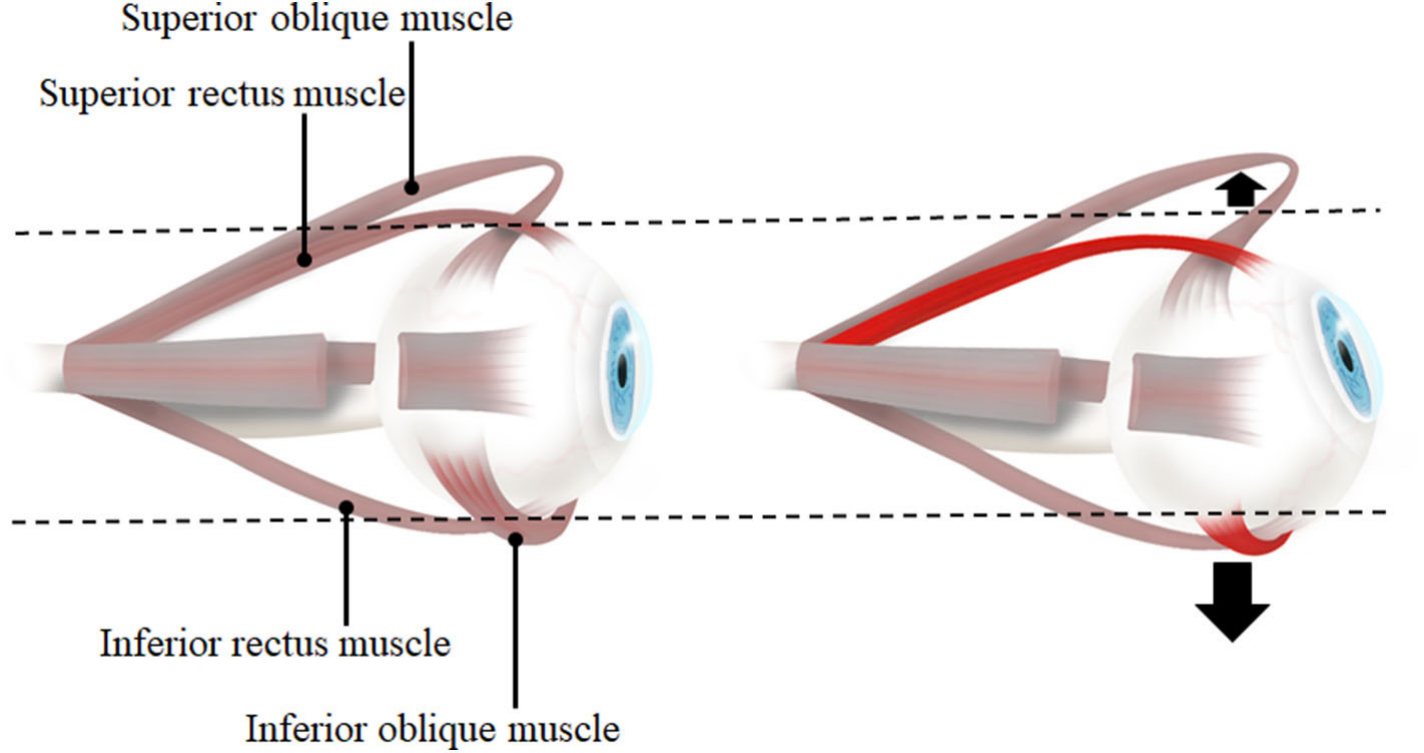
The lateral view of eye and extraocular muscles in primary position (left) and elevation (right). When the eye is elevated, the agonists are superior rectus muscle and inferior oblique muscle. Therefore, inferior oblique muscle which is inferiorly located to the eyeball also contracts in addition to superior rectus muscle. With the contraction of two muscles on opposite sides each other, the force to move the eyeball inferiorly would be larger than the force to superior side so that the direction of eyeball translation is downward. When the eye is depressed (not presented in this figure), inferior rectus muscle and superior oblique muscle contract and it would result in a net force to bring the eyeball upward.

Within our results, it is exciting to observe the differences in the direction of translation between horizontal and vertical gazing. During horizontal gazing, the translation is in the same direction as the gaze, whereas in vertical gaze it is opposite to the gaze direction. This difference with respect to gaze direction is due to the mechanics of the EOMs and pulleys (12, 13). In horizontal gaze, eye movement is only generated by the lateral and medial rectus muscles, whereas in vertical gaze, both the rectus and oblique muscles are involved (the superior rectus and inferior oblique muscles in upgaze, and the inferior rectus and superior oblique muscles in downgaze) (Figure 9). The horizontal rectus muscles are purely used for abduction and adduction, whereas the vertical rectus and oblique muscles are used for complex movements including elevation, depression, torsion, adduction, and abduction (28, 29). Moreover, the insertions of the oblique muscles are located behind the equator of the eyeball, unlike those of the rectus muscles. Therefore, due to the direction of action of the EOMs and pulleys, the eyeball translates in the same direction in horizontal gaze. Meanwhile, due to the complexity of the EOMs that act in vertical gaze, the translation direction in this situation is opposite to that of the gaze. Further studies exploring the mechanics of the vertical rectus and oblique muscles are needed to explain the opposite translation of the eyeball during vertical gazing.

Additionally, we found a negative correlation between the degree of translation and the size of the eyeball. Anatomically, the eyeball is surrounded by the orbital fat and it can therefore be assumed that it floats freely in the orbit (10, 30). The longer the axial length or the larger the eyeball volume, the greater the surface area of the eyeball that is in contact with the surrounding soft tissues. Considering our findings regarding the contact mechanics of the eyeball and the orbital fat, the contact pressure on the orbital fat is related to the contact area of the eyeball (31, 32). Although there are individual differences in the force applied by the EOMs and the mechanical properties of the orbital fat, it is generally the case that the smaller the eyeball, the higher the contact pressure, and the greater the translation that is possible within the orbital fat. These findings suggest that translatory movement should be considered, especially for small-eyed subjects such as children and far-sighted patients.

There are several limitations of this study. First, the MRI section thickness may have affected the measurement of ocular displacement. In particular, the mean distance of centroid movement in vertical gaze was <0.6 mm, the sectional thickness. However, by coordinating the centroid's position through the use of 3D reconstruction, the distance of centroid movement could be measured quantitatively. Second, due to the time taken for MRI, images could not be obtained from a range of angles. A study to determine the degree of translation according to the actual rotational angle is necessary, and further analysis of the correlation between the amount of rotation and translation for a large number of subjects is also needed. Third, MRI scanning were performed in prescribed experimental setting, not a physiologic state. Therefore, we were only able to obtain MR images for horizontal gaze and vertical gaze at preset angle, and MRI scanning were conducted with a fixed sequence in every patients: central gaze, right gaze, left gaze, upgaze then downgaze. Therefore, we could only evaluate the positional change of eyeball in a certain condition. And the fixed order of MRI scanning can induce some systematic errors or crossover effect in measurement. We should especially pay attention in analysis for images of vertical gazes because they were taken later in the MRI scanning procedures. In addition to a finite number of gaze directions and a fixed MRI scanning sequence, we were only able to use the target at about 30 cm and the fixation at near target could induce convergence. The actual eyeball rotation angles would be affected by the convergence. However, in our experimental setting, the effect of convergence on the rotational angle would be <1°. Therefore, convergence effect could be disregarded. Forth, MRI can only visualize the eyeball in static images, not in dynamic states. Therefore, we can only compare the eyeball position in different gazes, not evaluate the eyeball movement in real-time. Finally, as this was a study of healthy subjects, we cannot apply our results directly to patients with eye movement disorders such as strabismus. Future studies involving strabismic patients will therefore be necessary.

In conclusion, eye movement does not merely consist of rotation, but also translation, and translation occurs in all gaze directions. Additionally, the eyeballs translate in the same direction during horizontal gazing but in the opposite direction during vertical gazing. The fact that eye movement is a combination of rotational and translatory movement indicates that translatory eyeball movement should be considered in accurate eye movement analyses, such as in the measurement of deviation angles.

## Data Availability Statement

The datasets presented in this article are not readily available because the authors were not able to obtain the consent for the individual data disclosure from all study subjects. The result of the analysis is described for total data.

## Ethics Statement

The studies involving human participants were reviewed and approved by Institutional Review Board of Hanyang University Hospital. The patients/participants provided their written informed consent to participate in this study.

## Author Contributions

SO and HL contributed to the concept/design of the work. WL, SS, JL, and HL conducted the acquisition of data. YM, WL, JK, and HL contributed to the data analysis, interpretation, and drafted the intellectual content. All authors revised the final version.

## Conflict of Interest

The authors declare that the research was conducted in the absence of any commercial or financial relationships that could be construed as a potential conflict of interest.

## Supplementary Material

Clip 1 The adjustment of MR images based on the position of static tissues. We confirmed that only the eyeball and adjacent structures showed movement, not the brain or skull. Consecutive MR images represent the actual positional change of the eyeball in the axial plane.
